# The National Pension Fund

**Published:** 1888-01-14

**Authors:** 


### THE NATIONAL PENSION FUND.

WE have great pleasure in stating that as we go to press the announcement reaches us that £20,000 has been provided on the basis of Mr. Burdett's scheme to enable the necessary deposit to be made with the Master in Chancery. This Fund is therefore established at last on a firm financial basis. It is not possible to say more now, but we hope to be able to give full details in a later edition.

